# A Summary of New Findings on the Biological Effects of Selenium in Selected Animal Species—A Critical Review

**DOI:** 10.3390/ijms18102209

**Published:** 2017-10-21

**Authors:** Bozena Hosnedlova, Marta Kepinska, Sylvie Skalickova, Carlos Fernandez, Branislav Ruttkay-Nedecky, Thembinkosi Donald Malevu, Jiri Sochor, Mojmir Baron, Magdalena Melcova, Jarmila Zidkova, Rene Kizek

**Affiliations:** 1 Department of Viticulture and Enology, Faculty of Horticulture, Mendel University in Brno, Valtická 337, CZ-691 44 Lednice, Czech Republic; bozena.hosnedlova@post.cz (B.H.); jiri.sochor@mendelu.cz (J.S.); mojmir.baron@mendelu.cz (M.B.); 2 Department of Biomedical and Environmental Analyses, Faculty of Pharmacy, Wroclaw Medical University, Borowska 211, 50-556 Wroclaw, Poland; zalewska.m@gmail.com; 3 Central Laboratory, Faculty of Pharmacy, University of Veterinary and Pharmaceutical Sciences Brno, Palackeho 1946/1, 612 42 Brno, Czech Republic; sylvie.skalickova@gmail.com (S.S.); brano.ruttkay@seznam.cz (B.R.-N.); 4 School of Pharmacy and Life Sciences, Robert Gordon University, Garthdee Road, Aberdeen AB107GJ, UK; c.fernandez@rgu.ac.uk; 5 Department of Physics, University of the Free State, P. Bag X13, Phuthaditjhaba 9866, South Africa; malevu.td@gmail.com; 6 Department of Biochemistry and Microbiology, University of Chemistry and Technology, Technicka 3, 166 28 Prague, Czech Republic; magdalena.melcova@vscht.cz (M.M.); Jarmila.Zidkova@vscht.cz (J.Z.)

**Keywords:** selenium, antioxidant, oxidative stress, ruminants, horses, donkeys, metallomics

## Abstract

Selenium is an essential trace element important for many physiological processes, especially for the functions of immune and reproductive systems, metabolism of thyroid hormones, as well as antioxidant defense. Selenium deficiency is usually manifested by an increased incidence of retention of placenta, metritis, mastitis, aborts, lowering fertility and increased susceptibility to infections. In calves, lambs and kids, the selenium deficiency demonstrates by WMD (white muscle disease), in foals and donkey foals, it is associated with incidence of WMD and yellow fat disease, and in pigs it causes VESD (vitamin E/selenium deficiency) syndrome. The prevention of these health disorders can be achieved by an adequate selenium supplementation to the diet. The review summarizes the survey of knowledge on selenium, its biological significance in the organism, the impact of its deficiency in mammalian livestock (comparison of ruminants vs. non-ruminants, herbivore vs. omnivore) and possibilities of its peroral administration. The databases employed were as follows: Web of Science, PubMed, MEDLINE and Google Scholar.

## 1. Methodology of the Review

This review is focused on the biochemical and molecular genetic nature of selenium, on its physiological effects in mammalian livestock, on the possibilities of determining its status in the organism and on the importance of its additon to animals. The methodology of the choice used scientific studies from more than 2500 viewed articles based on the search phrases, such as: physiological effects of selenium, antioxidant/antibacterial/anticancer effect of selenium, selenium and oxidative stress/*Staphylococcus aureus*/immunity/thyroid hormones/reproduction, fertility/milk/rumen fermentation/gastrointestinal tract, glutathione peroxidase, epigenetic effects of selenium, selenium status analysis, selenium and analytical methods, selenium and cattle/sheep/goats/pigs/horses/donkeys; and the main findings of various studies were compared.

## 2. Biochemistry of Selenium

### 2.1. Importance of Selenium for Animal Health

Selenium (Se) is an essential trace element [1,2] that evinces antioxidant activity [2,3,4,5,6,7,8,9,10,11,12,13,14], anti-inflammatory [2,15,16,17,18,19,20,21,22,23,24,25], antimutagenic [26,27], anticarcinogenic [28,29,30,31,32,33,34,35,36,37] or chemopreventive [2,38,39,40,41,42,43,44], antiviral [2], antibacterial [45,46,47,48], antifungal [49,50] and antiparasitic effects [51,52,53]. Furthermore, it is an integral component of selenoproteins participating in a whole series of physiologically important processes [54]. The first proven selenoenzyme was glutathione peroxidase (GPx) [55], which is an indispensable component of the antioxidant system in the organism [56]. The selenoprotein family includes at least 25 eukaryotic proteins, whose expression is characterized by high tissue specificity, which depends on selenium availability and can be regulated by hormones [57,58,59,60]. Selenoproteins contain in their active site selenium in the form of 21st amino acid selenocysteine [61,62]. The incorporation of selenocysteine into selenoproteins utilizes an unique mechanism that requires decoding of the codon UGA [63] in mRNA, normally involved in the termination of translation [64,65,66]. The UGA recoding is strictly dependent on an RNA stem-loop structure, the selenocysteine insertion sequence (SECIS), which is found in the 3′ untranslated region (3′ UTR) of eukaryotic selenoprotein messages [67,68].

**Figure 1:**
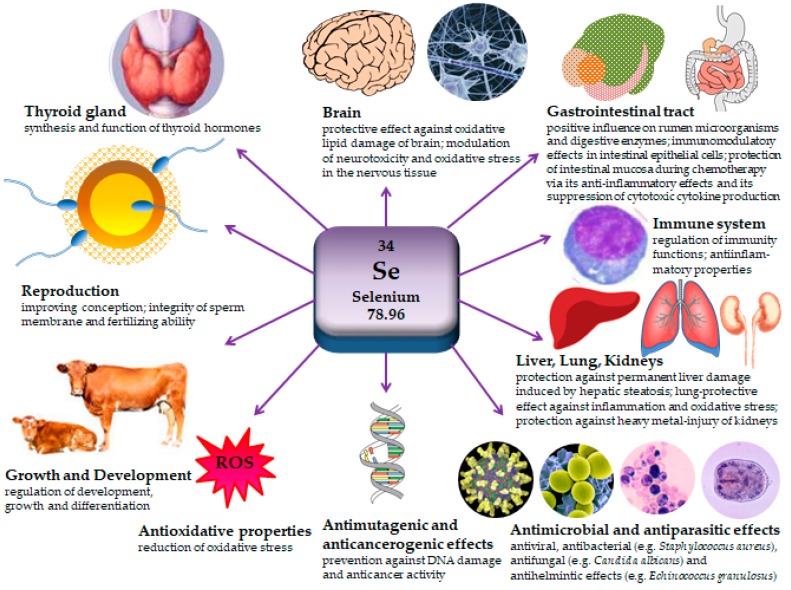
Physiological effects of selenium. Selenium is known for its antioxidant, antimutagenic and anticarcinogenic properties, it also acts against microbes as well as parasites and has antiinflammatory effects, engages in metabolism, growth and development, protects organs from oxidative stress, affects immune function and improves fertility [2,47,48,49,50,53,54,60,82,87,104,126,127,128,129,130,131,132,133,134,135,136,137,138].

Selenium is important for the synthesis [2,69], metabolism [63,69,70,71] and function of thyroid hormones [72], that are crucial regulators of development, growth and differentiation. In addition, they are also involved in many other physiological processes [54]. Selenium is a component of enzymes deionidases [73], which are divided into three types (D1, D2 and D3) and have different tissue distribution, gene expression regulation and function. D1 is primarily expressed in liver, kidney and thyroid [74,75] and is able to deiodinate thyroxine (T4). It is also essential to provide triiodothyronine (T3) for the circulation as well as serving as a scavenger enzyme for iodide in peripheral tissues [74]. D2 is expressed in a large number of tissues, such as skeletal muscle, bone, pituitary, retina, cochlea [76], CNS, thyroid and brown adipose tissue [75] and converts T4 into more active T3 [73] by 5′-deiodination [77]. In contrast, D3 inactivates T3 and, to a lesser extent, prevents T4 from being activated [78]. The level of T3 in blood increases with higher selenium intake [79,80]. The thyroid hormones, on the other side, affect directly the metabolism of selenium and its serum status as well as regulating the expression of some selenoproteins [81]. The physiological significance of selenium is shown in Figure 1.

Selenium is also important for the regulation of immunity functions [82], plays an essential role in non-specific immune response [83] and its low level is related to weakened immune system [84]. In inflammatory diseases, the selenium concentration declines and the biosynthesis of selenoproteins is disturbed [85]. The application of selenium decreases inflammatory activity [82]. Selenium is very important for chemotactic and phagocyte activity and respiratory burst activities. Selenium deficiency leads to GPx enzyme activity decreasing and the drop in neutrophil activity [86] as well as the cells becoming more susceptible to oxidative damage.

Selenium is involved in growth and development, as well as taking part in the regulation processes related with production [87,88,89,90,91,92,93,94,95,96,97,98,99,100,101,102,103], and reproduction abilities of animals [104,105,106,107,108,109,110,111,112,113,114,115,116,117,118,119,120,121,122,123,124,125].

### 2.2. Biochemical and Molecular Biological Activities of Selenium in Organism

Selenium is a metalloid with the atomic number 34 which belongs to the group 16 in the periodic table. It was discovered in 1817 by the Swedish chemist Jöns Jacob Berzelius. Selenium has six valence electrons, two of them being unpaired ([Ar]3d^10^4s^2^4p^4^). However, due to 4d orbitals, it is capable of formation of six covalent bonds. In oxygen-containing compounds it possesses +6, +4, and +2 oxidation states. With the majority of other elements, it forms binary compounds with an oxidation state of −2, e.g., in hydrogen selenide (H_2_Se) and organic selenides such as selenomethionine (SeMet) [127].

#### 2.2.1. Role of Selenium in Oxidative Stress

**Figure 2:**
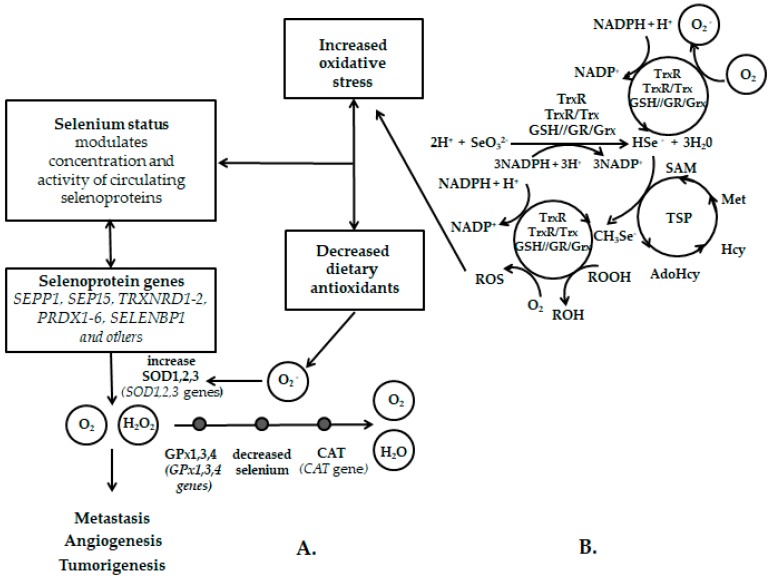
Biochemical and molecular biological scheme of selenium action (adapted from [139,140])—(**A**) Involvement of selenoprotein genes in the antioxidant metabolic pathway with modification of the selenium effect on the risk of carcinogenic process; (**B**) Spontaneous methylation of selenide to methylselenol. *SEPP1*—selenoprotein P gene; *SEP15*—selenoprotein 15 gene; *TRXNRD1–2*—thioredoxin reductase 1/2 genes; *PRDX1–6*—peroxiredoxin 1–6 genes; *SELENBP1*—selenium binding protein 1 gene; SOD1,2,3—superoxide dismutase 1,2,3; *SOD1,2,3*—superoxide dismutase 1,2,3 genes; O_2_—dioxygen; H_2_O_2_—hydrogen peroxide; O_2_^−^—superoxide anion; GPx1,3,4—glutathione peroxidase 1,3,4; *GPx1,3,4*—glutathione peroxidase 1,3,4 genes; CAT—catalase; *CAT*—catalase gene; NADP^+^—nicotinamide adenine dinucleotide phosphate; NADPH—reduced form of NADP^+^; TrxR – thioredoxin reductase; Trx—thioredoxin; GSH—reduced glutathione; GR—glutathione reductase; Grx—glutaredoxin; SeO_3_^2−^—selenite; HSe^−^—hydrogen selenide ion; CH_3_Se—methylselenol; SAM—S-adenosylmethionine; AdoHcy—S-Adenosyl-homocysteine; Hcy—homocysteine; Met—methionine; TSP—transsulfuration pathway.

Up to now, many genes that are related to the antioxidant properties of selenium have been identified: *SEPP1*, *SEP15*, *TRXNRD1–2*, *PRDX1–6*, *SELENBP1*, *CAT, GPx1,3,4*, *SOD1,2,3* (Figure 2A) [139]. The enzyme superoxide dismutase contributes to the reduction of oxidative stress by efficiently quenching the superoxide radical and converting it into less toxic hydrogen peroxide which the subsequent enzyme catalase (CAT) breaks down into water and oxygen to prevent DNA damage. In the case of reduced dietary intake of selenium and other antioxidants, its activity decreases and free radicals lead to the process of tumorigenesis. The cancer cells are shown to have increased reactive oxygen species (ROS) levels in comparison to their normal counterparts [129].

#### 2.2.2. Relationship of Selenium to Cancer 

Selenium exhibits toxicity against cancer cells [141]. It was reported that selenite stimulates apoptosis in cultured cancer cells [142,143,144,145,146], showing greater toxicity towards malignant compared to benign cells [147,148]. This also inhibits the development of mammary tumours in rat cells [149]. The anticancer action mechanism of selenite is based on the induction cancer cell death and apoptosis by producing superoxide radicals especially in or adjacent to mitochondria. Superoxide radicals trigger the mitochondrial pathway of apoptosis. It was found that selenite-induced superoxide production, cell death, and apoptosis were inhibited by overexpression of manganese superoxide dismutase (MnSOD; SOD2). The levels of MnSOD in cancer cells may influence the efficacy of administered selenium in cancer chemoprevention. Since cancer cells usually have lower levels of MnSOD, they should be more sensitive to selenium than their normal cell counterparts. Therefore, in cancer prevention, selenium may be found to selectively induce apoptosis of cancer cells without causing significant damage to normal cells [150]. Other researchers have also shown that selenite treatment damages mitochondria, leading to cell death [141,151].

With regards to other antioxidant enzymes, Xiang et al. [150] found that overexpression of Cu/Zn-SOD (SOD1), CAT and GPx1, in contrast to SOD2, did not suppress apoptosis in selenite-treated prostate cancer cell, while Kim et al. [152] reported that overexpression of SOD1 enhanced cell viability in malignant glioma cells exposed to selenite. The different effects of selenite on SOD1/2 expression, and SOD1/2 overexpression on selenite mediated cell death may be due to the multiple pathways by which selenite treatment induces ROS generation. Thus, a picture of the response of superoxide dismutases to selenite supplementation seems to be inconsistent [141].

The individual role of selenite, thioredoxin and the system of glutaredoxin and S-adenosylmethionine (SAM) in the redox cycle of selenium intermediate metabolites is shown in Figure 2B. The selenite is reduced to hydrogen selenide by thioredoxin, or glutaredoxin system. This reaction can also be catalysed by glutathione or cysteine to produce the same final products. Hydrogen selenide can be sequentially oxidized to a superoxide radical or undergo a redox cycle mediated by thioredoxin or the glutaredoxin system [153,154]. Hydrogen selenide can spontaneously react with SAM to form methylselenol (CH_3_Se). Subsequently, the thioredoxin and glutaredoxin system participate in the redox cycle of methylselenol with hydrogen selenide and generate reactive oxygen species. Under reductive conditions, monomethylselenol can, thanks to its extraordinary nucleophilicity, be compared to its counterpart hydrogen selenide and act as a free radical scavenger [140].

#### 2.2.3. Antioxidative Role of Selenium against the Toxic Effect of Heavy Metals

In the metabolic pathways of selenium, numerous proteins, including metallothioneins (MTs), [155], which play a role in heavy metal detoxification, are involved. The exposure to heavy metals, e.g., mercury (Hg) is often associated with the induction of metallothioneins [156]. Intraperitoneal selenium administration to rats exposed to mercury resulted in a complete recovery of mercury-altered levels of oxidative stress parameters, to returning of the mercury-induced mRNA expression levels of *MT-I* (metallothionein-I) and *MT-II* (metallothionein-II) in the liver to their original state, and had also partial protective effect on the kidneys [157]. Selenium is considered to be an antidote in the treatment of mercury intoxication [158], it may modulate Hg toxicity through the influence on Hg deposition in tissues, as well as Hg-induced oxidative stress, inflammation, excitotoxicity, and other pathways [127]. Studies by Orct et al. [135] demonstrated the protective effect of selenium on oxidative lipid damage in the brain, which is extremely sensitive to mercury during the early postnatal period. Severe selenium deficiency causes irreversible brain injury [159]. Experimental data demonstrate that selenium treatment modifies brain mercury retention, and modulates neurotoxicity and oxidative stress in the nervous tissue of animals [127]. Selenium counteracts the neurotoxicity of mercury presumably through the formation of nontoxic complexes [160]. The circulating selenium transporter, selenoprotein P (SEPP), appears to have a special role in the delivery of selenium to the brain and neurons by entering via the multifunctional apolipoprotein E receptor 2 (ApoER2), a member of the lipoprotein-receptor family that is expressed in neurons in the brain [161].

A protective effect of selenium was also reported against toxicity of other heavy metals, such as chromium (against chromium-induced oxidative and cellular damage in thyroid gland [162], chromium toxicity in the brain [163] and chromium-induced nephrotoxicity [164]), cadmium (the protection of jejunal epithelial cells from cadmium-induced DNA damage [165], an antagonistic effect of selenium on cadmium-induced damage of kidney [166], ameliorative effect against cadmium chloride-induced neuro- and nephrotoxicity [167]), and lead (against lead toxicity on the antioxidant system [168]), or also overabundance of iron. Selenium reduces the adverse impacts of excess iron intake. Iron is important for many biological processes, it is distributed to the cells bound to serum protein transferrin and the iron transport into the cells takes place via the internalization of the transferrin bound to the transferrin receptor. The expression of the transferrin and ferritin genes is strictly regulated by the iron responsive element (IRE) and the iron regulatory protein (IRP) [169]. Free iron generates ROS that damage lipids, proteins and DNA [170]. Chronic iron excess was associated with a decrease in selenium concentration and GPx activity in the heart tissue in mice, and the addition of Na_2_SeO_3_ to mice reduced the concentration of iron in the heart tissue and oxidative stress [171,172].

#### 2.2.4. Epigenetic Effects of Selenium and Their Implications for Prevention of Carcinogenic Process

Selenium has been found to modify epigenetic marks [65]—mitotically stable chromatin-based mechanisms that modulate gene expression without altering the genomic DNA sequence. These mechanisms include modifications to DNA and to histones (acetylation, methylation and many others) [173], which interfere with chromosomal packaging and the binding of trans-acting factors [174]. Changes in the epigenome are associated with a great variety of diseases [175,176,177], including also inflammatory disease [178] or the onset and progression of carcinogenic process [179,180].

The role of selenium for health is based on its biological functions that are presented by the members of the selenoprotein family encoded by more than tens of genes and containing cotranslationally inserted selenocysteine [65]. It is also a non-selenoprotein pool of low-molecular-weight selenium compounds contained in the diet or derived from selenium metabolism [174]. The importance of selenoproteins for development and health has been clearly demonstrated in transgenic mice with single [181] or total [182] selenoprotein depletion.

Research studies performed on rats [37,183,184], mice [185] and cell lines [186] have shown that selenium intake affect global DNA methylation. However, the rodent studies gave inconsistent results regarding an increase or decrease of global DNA methylation in response to dietary selenium [174].

In mouse embryonic stem cells (ESCs), after their exposure to selenium, was found a reversible alteration of the cell heterochromatin status and also the changed DNA methylation status of genes with crucial roles in fetal development, such as *Aebp2* (AE binding protein 2), *Prickle2* (prickle homolog 2) and *Rnd2* (Rho family GTPase 2), without compromising cellular potential for embryonic development. This implies that the genes with various functions regulated by DNA methylation are affected in ESCs as an in vitro model for early embryos [187].

Selenium deficiency resulted in less DNA methylation in rat liver [184] and colon [183] in contrast to the later study, that found significantly less global liver genomic DNA methylation in rats with supranutritional dose of selenium than those fed with selenium-deficient diet. These differences could be due to various rat inbred strains and selenium content of the basal diets as potential modifiers of selenium effects [188]. Another possibility to influence the differences [174] is using various techniques applied for the assessment of global DNA methylation [185].

Besides of global genomic DNA methylation, the regulation of site-specific DNA methylation of tumor suppressor genes is important and it has been considered as a leading mechanism by which some nutrients exert their anticancer property [188]. Alterations in DNA methylation, which are associated with DNA methyltransferase abnormalities and result in silencing of tumor-related genes and chromosomal instability, are involved in precancerous changes in various organs [179]. The study whether selenium affects the methylation of the *p53* gene was investigated, and it was found that supranutritional dose of selenium significantly increased the exon-specific DNA methylation of the *p53* gene (in exons 5–8) in liver and colon mucosa of rats compared with this in animals fed with the selenium-deficient diet [188].

Selenium has been shown to be associated with changes to histone marks [174]. Interference of selenium with histone marks can principally occur through modulation of histone modifying enzyme activity/expression and via interference with substrate availability. In consideration of the large variety of marks and participating enzymes, the situation is even more complex than for DNA methylation; moreover crosstalks exist between DNA methylation and histone marks, thus a complicated network of epigenetic regulation is formed [189]. The abnormal function and/or expression of histone deacetylases (HDACs) is linked to cancer and some neurologic and immune disorders. Numerous synthetic HDAC inhibitors have been developed and are currently tested in clinical trials [190]. Studies have confirmed that dietary and synthetic selenocompounds inhibit HDAC activity [191,192,193].

Selenium also affects the microRNA (miRNA) expression. The regulation of gene expression by targeting of mRNA through non-coding RNA molecules such as miRNA is considered an additional epigenetic mechanism. Microarray analysis (comprising of 737 miRNAs in total) of the miRNA profiles of Caco-2 cells grown in selenium-deficient or selenium-supplemented medium revealed that the expression of 12 miRNAs was affected by selenium supply [194]. Expression levels of 50 mRNAs were also selenium-responsive, and numerous of the mRNAs were predicted to be targeted by the selenium-responsive miRNAs. One of these, miRNA-185, whose expression decreased under selenium deficiency, was confirmed to regulate expression of glutathione peroxidase 2 (*GPx2*) and selenophosphate synthetase 2 (*SPS-2*) genes. As the protein product of *SPS-2* is component of the selenoprotein biosynthesis process, it indicates that selenium intake affects the selenoproteome in part through epigenetic mechanisms involving miRNA-185 and possibly also other miRNAs. miRNA-185 is an especially interesting target of selenium, because it has been recently introduced as a tumor suppressor that is often downregulated in various type of cancer [195,196].

Finally, selenium has been shown to act anticarcinogenic in various epigenetic studies, but the research of the detailed role in cancerogenic process is still in its infancy, it still requires a lot of experimental studies in the future, especially it will be very interesting to reveal the role of miRNAs as mediators of selenium-dependent tumor protection against malignant transformation.

### 2.3. Health Disorders of Animals Associated with Selenium Deficiency

#### 2.3.1. Described Diseases Associated with Selenium Deficiency

Many selenium deficiency diseases are often referred to the lack of another important nutrient with antioxidant function such as vitamin E. The variety of the antioxidant defense system components of the body allows it to profit from different types of antioxidants. This is especially known in these two nutrients in the etiology of certain diseases in which a nutrition deprivation of either one or the other can be asymptomatic, whereas the deficiency of both causes the disease. For example, animals fed with low-selenium diets commonly require higher amounts of vitamin E than animals sufficiently supplemented with selenium [197]. In addition to their joint involvement in the antioxidant system, the presence of both for the proper function of the immune system and resistance against infections is just as important [198,199].

The known manifestation of selenium deficiency in calves [200,201], lambs, kids [201], foals [201,202] and donkey foals [203] is white muscle disease (WMD) or nutritional muscular dystrophy (NMD). The disease can be also caused by the lack of vitamin E or a combined lack of selenium and vitamin E. The clinical symptoms include stiffness, weakness and recumbency [201]. The disease involves hyaline degeneration of muscle cells in various skeletal muscles, including the diaphragm and the heart [204]. In ruminants, WMD is also manifested by changes in the frequency and quality of heartbeat [205]. In lambs with WMD, the arrhythmia was diagnosed using electrocardiography (ECG) [206]. The arrhythmia is observed at early stages of cardiomyopathy. The electrocardiograms of diseased calves revealed elevated heart rate, accelerated sinus rhythm, increased P wave amplitude, shorter PR, QT and ST interval, narrower QRS complex, shorter T wave duration and insignificantly increased T wave amplitude [205].

The deficiency of selenium and vitamin E in horses [207] and donkeys [203,208] caused a yellow fat disease or steatitis, provoking the degeneration of the adipose tissue that is replaced by connective tissue and calcium deposits, and can be associated with dystrophic myodegeneration (white muscle disease) [203]. The symptoms are inertia, recumbency, decreased appetite, weight loss, fever, ventral oedema, stiff gait and painful neck. It is also very common to find low hematocrit, decreased selenium and vitamin E concentrations as well as elevated levels of creatine kinase and lactate dehydrogenase [207].

In pigs, the deficiencies of selenium and vitamin E caused the so-called VESD (vitamin E/selenium deficiency) syndrome, of which the most frequent manifestation is mulberry heart disease, which also included *hepatosis dietetica* and nutritional myopathy [209].

#### 2.3.2. Effect of Selenium on Female Reproduction

The selenium administration in selenium-deficient cows can reduce the number of services per conception, improve the pregnancy rates at first service and result in fewer days to conception. The increase of selenium concentration prepartum in blood correlates with anoestrus/silent oestrus decreasing postpartum [104]. The selenium deficiency caused abortions [111,210] and stillborn [211]. Giadinis et al. [111] reported that the selenium deficiency in grazing beef cattle was the sole cause of abortion in cows. The findings of lesions on fetal heart and skeletal muscle were consistent with WMD. The most probable mechanism of the abortifacient effect of selenium deficiency is, among others, fetal heart failure [212]. Another mechanism could be an insufficient progesterone concentration required to maintain the pregnancy in the late gestation [210]. The administration with selenium to pregnant cows contributes to adequate progesterone secretion [210] and also promotes its postpartum production [213].

The selenium and vitamin E administration reduces the incidence of retained placenta [121]. Selenium also has an effect on the decrease in the incidence of metritis and ovarian cysts [214]. The significant increase in the expression of the glutathione peroxidase 1 gene (*GPx1*) in granulosa cells of healthy follicles points to antioxidant role of GPx1 during the ovarian follicular development [215].

#### 2.3.3. Effect of Selenium on Male Reproduction

Antioxidant protection plays a key role in maintaining the integrity of the sperm membrane and their fertilizing ability [60]. Selenium involved in the antioxidant defense of the organism substantially modulates the quality of the male ejaculate [123]. In testes, several selenoproteins such as selenophosphate synthase (SPS-2) and mitochondrial capsule selenoprotein (MCSeP) were localized [216,217]. Oxidative stress (OS) is an important factor that negatively affects the fertility potential of spermatozoa by lipid peroxidation [218]. Sperm plasma membrane is extremely susceptible to lipid peroxidation due to the presence of high concentration of polyunsaturated fatty acids (PUFAs) [219,220,221]. Those PUFAs give to the membrane a high level of fluidity and elasticity necessary for sperm motility and their fusion with oocytes. Lipid peroxidation can lead to loss of membrane fluidity and integrity, and thus to reducing of sperm-oocyte fusion ability [221]. The number of spermatozoa and their motility are fundamental indicators of sperm functional ability [218].

Under physiological conditions, ROS are key for sperm function [222]. In small amounts, they are necessary for fertilization, acrosomal reaction, hyperactivity, motility and capacitation [223]. Under pathological conditions, however, excessive ROS levels may negatively affect their quality [222]. Spermatozoa, as cells living in aerobic conditions, face an oxygen paradox: O_2_ is vital for them, but its metabolites, such as ROS, can alter sperm functions and endanger their survival. ROS cause an infertility with two key mechanisms. Besides sperm membrane damage, which results in decreased sperm motility and their ability to fuse with oocytes, they can also damage sperm DNA, leading to the transmission of defective paternal DNA to fetus [108]. ROS may attack DNA by modification of nitrogenous bases, DNA strand breaks, DNA cross-links, and chromosomal rearrangements [221].

One of the markers of oxidative stress are the TBARS (thiobarbituric acid reactive substances) [224,225], of which the most widely used is malondialdehyde (MDA), the product of aldehydic lipid peroxidation generated by the action of ROS on membrane lipids [226]. MDA is one of the reactive and mutagenic aldehydic lipid peroxidation products in seminal plasma [227], and can be considered as a diagnostic tool in male infertility [228]. Breininger et al. [229] found a high negative correlation between TBARS and sperm motility in boars. An increased level of MDA in stored boar semen was associated with a rapid loss of motility and integrity of the plasma membrane of spermatozoa [230]. The reduction of motility may be due to ROS-induced impairment of ATP (adenosine triphosphate) utilization or contractile apparatus of the sperm flagella [231].

Reference values for MDA and other oxidative stress parameters in mammalian livestock are shown in Appendix Table A1.

#### 2.3.4. Effect of Selenium on Reduction of Intramammary Infection and Milk Quality

Selenium deficiency is also associated with an increased incidence of mastitis [60,232,233]. Dietary intake of selenium and vitamin E reduces their incidence [121]. The primary defense mechanism against mastitis is the phagocytic activity of neutrophils [233]. In dairy cows with selenium deficiency, the phagocytic ability of the blood and neutrophils decreased [234]. Selenium affects the innate and the adaptive immune responses of the mammary gland through humoral and cellular activities [235]. The dietary selenium intake at the dose of ≥4 mg∙day^−1^ prepartum was negatively associated with the likelihood of bovine intramammary infections due to coagulase-negative staphylococci [236]. The higher content of selenium in bulk milk tank samples was linked with the lower risk of *Staphylococcus aureus*-positive herd. Increasing bulk tank milk selenium by 0.2 μmol∙L^−1^ reduced the odds by a factor of 0.95 [237]. The effect of selenium on mammary pathogens is mediated by several mechanisms: a faster and more massive influx of polymorphonuclear leucocytes (PMN) into the udder [238], the more effective killing of bacteria (such as *S. aureus*) by PMN [239], high antibacterial activity of whey inhibiting growth of *S. aureus* [240] and high expression of selenoproteins with antioxidant properties in the mammary gland [241].

The administration of selenium to heifers and cows before calving reduced the prevalence of intramammary infections and high somatic cell count (SCC) during early lactation [242,243]. Injection application of Se together with Zn, Mn and Cu had a positive impact on reductions of somatic cell scores (SCSs) and mastitis incidences [244]. In goats, that were administered with selenium and vitamin E, significantly lower somatic cell counts were observed compared to the control group [101].

The dietary addition of selenium increases the selenium concentration and the percentage of favourable PUFAs and cis-9, cis-12 linoleic acid in cow’s milk [245]. In milk, most of the selenium is present in whey (47.2–73.6%) and least in the fat-phase of milk (4.8–16.2%) [246]. The dietary addition of selenium along with vitamin E increased the percentage of crude protein and lactose [247].

#### 2.3.5. Effect of Selenium on Rumen Fermentation

Selenium can influence rumen microbial fermentation. Administration of SeMet increased in vitro production of short-chain fatty acids (SCFAs) by rumen microflora. On the contrary, the effects of selenite and elemental selenium on the increasing amounts of these acids were not significant. The fermentation rate was faster in the presence of SeMet when compared to elemental selenium and selenite; the plateau of fermentation of SeMet was reached within 30 h, whereas for other two mineral forms it was not reached until at least 36 h. The ratio of *acetate:propionate:butyrate* was differently according to the selenium source. In the presence of SeMet, the increased proportion of acetate was observed, which could be explained by the probable utilization of SeMet by rumen bacteria as an energy source [248]. In case of limiting carbohydrate sources, proteins are degraded to NH_3_, amines, gas and SCFAs, and acetate predominantly produces fatty acids [249,250]. In the presence of selenite, there was found contrarily the increased proportion of butyrate [248].

Rumen microorganisms alter the bioavailability of received selenium [251]. Selenite or selenate delivery to ewes decreased whole blood and serum selenium concentrations compared with ewes receiving the same selenium dosage in form of SeMet as Se yeast [252]. Incorporation of selenium into microbial biomass was ex vivo greater for SeMet (13.2-fold higher than for control group) compared with inorganic selenium addition (selenite, selenate) [251]. A large amount of amino acids, which are released by microbial proteolysis in the rumen, are re-utilized for microbial protein synthesis. SeMet is not absorbed in situ to any appreciable extent in the rumen [253]. Bacteria reduce selenate (SeO_4_^2−^) through selenite (SeO_3_^2−^) to elemental Se (Se^0^). Selenium can also become incorporated into proteins as part of the amino acids selenocysteine (SeCys) or SeMet. This occurs as a result of the reduction of selenite with reduced glutathione to make selenodiglutathione, which is subsequently reduced to glutathioselenol (GS-SeH), which is further reduced to hydrogen selenide (H_2_Se) providing the necessary reactive intermediates for selenium incorporation into amino acids, or further reduction to elemental selenium [254]. When a high selenium diet is administered to beef cattle, the number of selenium-reducing microorganisms increases [255].

Dietary supplement of selenized yeast at the dose of 150–300 mg∙kg^−1^ (which provided 0.15‒0.30 mg Se∙kg^−1^) of feed dry matter to cows increased the volatile fatty acid (VFA) concentration and altered the rumen fermentation towards higher propionate production compared with the control group. The dose of about 300 mg∙kg^−1^ of dry matter of food stimulated the digestive microorganisms and enzymes [higher digestibilities of dry matter, organic matter, crude protein, ether extract, aNDF (amylase-treated neutral detergent fiber) and ADF (acid detergent fiber) in the total tract compared to the control group [87].

Supplementation of selenium to sheep influences the development of some ciliates: the use of selenized yeast has a more pronounced effect on *Ophryoscolex* and *Diploplastron* populations than the equivalent amount of sodium selenite. The first-mentioned genus is the most sensitive to selenium in the diet, and in sheep with its deficient intake it is not even found [256]. The selenium form also affects the enzymatic activity in the rumen fluid of the sheep. GGT (gamma-glutamyl transferase) and GDH (glutamate dehydrogenase) activities were significantly higher after administration of selenized yeast, AST (aspartate aminotransferase) and ALP (alkaline phosphatase) than when sodium selenite was used [257].

#### 2.3.6. Effect of Selenium on Hair Production

Selenium intake is important for the bioactivity of IGF-1 (insulin-like growth factor 1) [258], which stimulates the proliferation, migration and morphogenesis of hair follicle cells through specific cell-surface receptors (IGF-1 receptor, IGF-1R) during ontogenetic development [259]. IGF-1 represents peptide hormone produced in the liver, which is formed as a consequence of growth hormone (GH) release from the pituitary gland, which stimulates subsequently IGF-1 production in the liver. IGF-1 is therefore a mediator for some of the GH functions, thus involved in growth and anabolism. IGF-1 mediates its effects by binding at the specific receptor [260].

Wu et al. [102] reported that selenium administration to pregnant goats increases the antioxidant defense of fetal skin and improves the growth and development of fetuses and the development of their hair follicles by up-regulating IGF-1.

**Figure 3:**
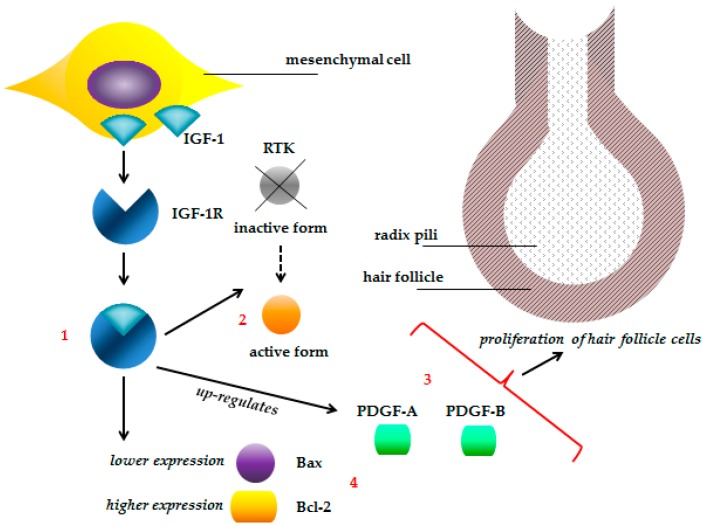
Role of IGF-1 in stimulation of hair follicle cell proliferation. Insulin like growth factor 1 (IGF-1) is produced by mesenchymal cells and binds to the insulin like growth factor 1 receptor (IGF-1R). This binding (**1**) activates the receptor tyrosine kinase (RTK) (**2**) [264], increases the expression of platelet-derived growth factors (PDGF-A, PDGF-B) (**3**) and the expression ratio of Bcl-2 (anti-apoptotic protein)/Bax (pro-apoptotic protein) (**4**), and thus stimulates the proliferation of hair follicle cells [262].

The growth of hair is a cyclic process in which every follicle proceeds from an active phase (anagen) through a regression phase (catagen) to a resting phase (telogen). During catagen, hair follicles undergo apoptosis and there is a decline in the level of an anti-apoptotic protein Bcl-2, and an increase in a pro-apoptotic protein Bax [261]. The effect of IGF-1 on hair growth appears to be related to the upregulation of PDGF-A and PDGF-B and to the anti-apoptotic effect of IGF-1 [262] (Figure 3). IGF-1 stimulates hair follicle growth, maintains the anagen stage and postpones the catagen stage by increasing the expression of the platelet-derived growth factors (PDGF-A, PDGF-B) as well as the expression ratio of Bcl-2/Bax [262]. PDGF is a potent mitogen produced in a variety of cell types including keratinocytes and endothelial cells, and is important for cell growth [263].

IGF-1 activates two signaling pathways: (1) the extracellular signal related kinase/mitogen activated kinase pathway and (2) the phosphatidylinositol 3′-kinase/protein kinase B (PI3K/Akt) pathway [262]. To exert its biological effects, IGF-1 must activate cells by binding to specific cell-surface receptors. The type I IGF receptor (IGF-1R) is the only IGF receptor to have IGF mediated signaling functions [265]. IGF-1 is produced by mesenchymal type cells and acts in a paracrine fashion and/or an autocrine fashion by binding to the IGF-1R. This binding activates the receptor tyrosine kinase that triggers the downstream responses and finally stimulates cell division [264]. IGF-1 may therefore be able to stimulate the proliferation of hair follicle cells through cellular signaling pathways of its receptors [262].

An in vitro study showed a significant difference in cumulative hair follicle elongation between the control group (0.97 ± 0.09 mm) and the 10^−7^ M IGF-1 treated hair follicles (1.24 ± 0.09 mm) over a period of 12 days [262]. On the 2nd day, the IGF-1 treated group showed more prominent expression of PDGF-A, and PDGF-B also showed a significant increase in expression. Among the apoptosis related molecules, Bax and Bcl-2 showed differences in expression. On the 8th day, Bax was weakly expressed in the IGF-1 treated group. On the 2nd day, Bcl-2 was more strongly expressed in the IGF-1 treated group compared with the control group [262].

## 3. Selenium Status Assessment in Animals

### 3.1. Selenium Status Assessment

The selenium status in the organism can be evaluated directly based on the determination of selenium content, or using the indirect method—based on the activity of selenium-dependent glutathione peroxidase. The reference values (RVs) are given in Appendix Table A1.

### 3.2. Total Selenium Concentration

The status of selenium can be assessed based on its content in blood, urine, tissues, excrements, and in lactating females in milk. Selenium content is usually detected by hydride generation-atomic absorption spectrometry (HG-AAS) [266,267,268]. The HG-AAS method can be used to determine the content of selenium in whole blood [266,269,270,271,272], blood plasma, serum [268,272,273] and also in tissues (e.g., liver, skeletal muscle, myocardium, kidneys) [272,274]. For all these samples (taken from cows) after wet mineralization using HNO_3_ and H_2_O_2_ and subsequent reduction by HCl, the detection limit (around 0.8 μg∙L^−1^) and the measurement errors (4.6–15%) of this method corresponded to the requirements for use in research and clinic as well as preventive veterinary medicine [272].

The selenium concentration in whole blood and milk can also be determined by the ICP-MS method (inductively coupled plasma-mass spectrometry) [275], which enables to determine the absolutely accurate concentration of the chemical element in samples compared to AAS, which is contrarily suitable for routine analysis with lower cost. The ICP-MS method is also usable for analyzing the total amount of selenium in feed samples [276,277], urine and faeces [277].

Selenium can be also analysed using ICP-OES (inductively coupled plasma-optical emission spectrometry), specially serum selenium [278] as well as analysing the selenium content in diet and tissue of muscle [279]. This method is considered to be less sensitive than ICP-MS. The fluorometric method has been succesfully employed to analyse selenium in feed [280,281], excrements, blood and tissues [281]. The FIA-GF-AAS (flow injection analysis-graphite furnace-atomic absorption spectrometry) system coupled with a hydride generation has been utilised to detect selenium in plasma and spleen samples [282]. VG-ICP-MS (vapor generation-inductively coupled plasma-mass spectrometry) has also been used as an analytical tool to analyse selenium content in feed, milk and whole blood [275].

The mere knowledge of the total concentration of the element in biological materials is not sufficient for the evaluation of its effects, it is also necessary to know the distribution between individual binding forms—species [283]. Data on the concentration of individual selenium species are obtained in the speciation analysis process [284], which is usually performed by the combination of separation methods and trace element analysis methods [285].

Selenium species are most commonly detected by the combination of liquid chromatography (LC) and ICP-MS—LC-ICP-MS [286,287], or the HPLC-ICP-MS [288,289,290,291,292,293]. HPLC (high-performance liquid chromatography) is also usable for detecting selenium species in plant feed [276]. Speciation selenium analysis is most often performed at the level of individual seleno-amino acids and other low-molecular substances: selenite (Se^+IV^), selenate (Se^+VI^), selenomethionine (SeMet), selenocystine (SeCys2), Se-methylselenocysteine (MeSeCys) and others, which are released into the solution by enzymatic hydrolysis by non-specific proteases [288,289,290,292,294,295,296,297]. Separation of these substances is performed by various LC modes, in particular ion exchange chromatography (IEC) [290,293,294,295,296,297], and reversed-phase chromatography (RPC) [288,289,292,293,298].

Serum or plasma selenium concentrations more accurately reflect the current level of supplementation and are more sensitive to short-term changes in selenium administration than its whole blood level, reflecting more of its earlier supplementation because selenium (in glutathione peroxidase) is incorporated into erythrocytes during their formation (erythropoiesis). The whole blood selenium responds slowerly to changes in supplementation (with supplementation increases more slowly, without supplementation decreases more slowly than serum or plasma selenium levels) [299].

Although different methods are used to determine the selenium content in blood, they seem to be comparable, e.g., the detection limit in ICP methods is similar to that of fluorometric method using 2,3-aminonaphthalene [300,301], in an interlaboratory study the mean concentration of selenium in the blood in cattle using a fluorometrical analysis was similar to that of HG-AAS method [302]. However, the concentration of selenium varies according to the sample type, there are the differences in its content in whole blood [270,303], serum [304,305] and plasma [102,306].

When HG-AAG and inductively coupled argon plasma emission spectroscopy using hydride generation (ICP) are compared, a relatively similar correlation coefficient between the content of selenium in whole blood and serum in cattle was found to be: 0.79 (*r*^2^ = 0.62) for HG-AAS, and 0.88 for ICP (*r*^2^ = 0.77 (simple linear regression model), and adjusted *R^2^* = 0.82 (expanded regression model)). However, the prediction intervals were relatively wide and the diagnostic accuracy for estimating blood selenium concentration based on its serum level is therefore very limited. For example at serum values of 0.01–0.05 μg∙mL^−1^, the predicted selenium concentration in blood ranged from a significant deficiency (<0.05 μg∙mL^−1^) to a normal value (>0.10 μg∙mL^−1^). Measurement of serum selenium level may not indicate whether blood selenium is within the reference range or at toxicity level. For diagnostic and clinical use, serum selenium levels must allow to distinguish normal selenium status from its deficiency. Approximately 67% of the normal distribution of the population could use the ratio *blood selenium:serum selenium* of 1.6–3.4:1 for data obtained from HG-AAG, and 1.1–3.4:1 for ICP. Practical use of these ratios for diagnostic purposes, whether blood selenium is in the reference range or deficiency range, is not appropriate. This fact cannot be determined based on serum selenium concentrations. The reason for the failure of this methodology is probably the variability of the hemolysis rate between the samples, and as a result of this, selenium releases from the erythrocytes into serum. Thus, a short-term change in selenium intake in the diet, reflected in blood serum but not yet in the erythrocyte selenium concentration (incorporation of selenium into erythrocytes lasts from days to weeks). In addition, genetic variability could influence selenium metabolism and the relationship between selenium in serum and blood [307].

The concentration of selenium in blood or serum is influenced by the selenium content in the diet [114,308,309,310,311] and by form of received selenium [309,312]. The content of selenium in blood also differs according to the breed—Jovanović et al. [270] vs. Pavlata et al. [267] (Appendix Table A1), sex [313] and the age of the animal [314] as well as geographical area, which is related to the selenium content in the soil and thus also in plants [315,316]. Generally, most soils in many countries are poor in selenium [317,318,319,320], sometimes even below 0.2 mg∙kg^−1^ [321]. The soil selenium concentration depends on soil-climate interactions. The low-selenium soils are most likely to occur in arid regions and in areas with high pH and low clay content. Conversely, the areas with low to moderate precipitation but relatively low aridity (e.g., cool and moist climates) and high clay content are likely to have higher soil selenium concentrations [322]. In addition to the total soil selenium content, soil physical-chemical conditions are also important, because can cause lower bioavailability of selenium and thus its lower uptake by plants, which can ultimately lead to its deficiency in animals [321]. For example, the negative influence of sulfate (SO_4_^2−^) and phosphate (PO_4_^3−^) has been recently described [323]. The selenium bioavailability in soil is influenced also by the presence of organic acids, important components founded in the rhizosphere soil [324].

The reference values of selenium (Appendix Table A1) in blood are different depending on the species and age of the animal, but also according to the used author’s methodology, for example Constable et al. [304] stated a range of 0.08–0.30 μg∙mL^−1^ for blood serum in adult cattle, while Stowe and Herdt [314] published a 3-fold lower upper limit (100 ng∙mL^−1^). However, many authors use the value of the lower limit of RV, according to different publications, ranging from 50 to 100 μg∙L^−1^ [269,314].

### 3.3. Enzymatic Methods of Assessment of Selenium Status

Besides the direct detection of selenium status by determining its concentration in blood, an indirect method based on glutathione peroxidase activity assessment can be used. Approximately 11.8% of total selenium in the body is bound to this protein [325]. GPx contains, with the exception of phospholipid-hydroperoxide GPx (PHGPx), which is monomeric [326], four selenium atoms and positively correlates with the amount of selenium in blood [269]. The high correlation coefficient between selenium and GPx activity in whole blood was found to be *r* = 0.82 [269], *r* = 0.93 [313], *r* = 0.97 [327], respectively. Despite this significant correlation between these parameters, the relationship between their values reported in various publications is often very inconsistent [269], e.g., Hogan et al. [328] presented a blood selenium value in cows of 270 μg∙L^−1^ and a corresponding GPx activity of 80 IU∙L^−1^, while Ellison [329] reported a concentration of 19.75 μg∙L^−1^ selenium and GPx activity of 2000 IU∙L^−1^ as the lower limit of the physiological range.

GPx activity depends on selenium content in the diet [303] and is positively correlated with the selenium intake [330]. This is due to the fact that most of the glutathione peroxidases (GPxs) (GPx1, GPx2, GPx3 and GPx4) contain selenocysteine at their catalytic site and therefore the activity of these enzymes is dependent on the accessibility of selenium [331]. Thus, the measurement of seleno-dependent glutathione peroxidase activity (SeGPx) in blood is a widely used indicator of selenium status [332,333], antioxidative status and potential [334,335], as well as a parameter for evaluating the response to its therapeutic administration [174,336]. However, GPx activity response to dietary selenium intake is very inconsistent in various studies [114,337,338,339,340]. Although GPx activity is a useful biomarker of selenium status for a particular individual, substantial heterogeneity can be observed in various publications [341]. Appendix Table A1 also shows a considerable variability in values. This is due to the use of various measurement units and, in particular, by a wide variety of analytical methods used [269]. The authors do not indicate enzymatic activity in blood only in μkat∙L^−1^ of whole blood [270], but also in μmol∙g^−1^ of hemoglobin (Hb) [304], U∙g^−1^ of hemoglobin [342], mmol∙L^−1^ of serum [343], IU∙L^−1^ serum [344], U∙mL^−1^ of serum [102]. There is a vast variety of other reference units found in the literature such as: IU∙L^−1^ of whole blood, IU∙mL^−1^ PCV (packed cell volume) [269], per mg of blood plasma protein, mg of hemoglobin, mL of whole blood as well as mL of blood plasma [345]. Different units appeared even at defined reference limits in various countries, the activity value also depends on the chosen method of calculation (linear vs. polynomial regression): RV for sheep bred in Iran 191.67–196.52 U∙g^−1^ of Hb [346] vs. in the Czech Republic >637 μkat∙L^−1^ (LR), resp. 677 μkat∙L^−1^ (PR) of whole blood [271]. For this reason, literary data can usually not be compared. The individual methods of analysis differ in different reaction temperatures in vitro and in the use of various anticoagulants [269]. The evident differences in RV determination are even reflected within the same author for one animal species in the same state (RV for GPx activity in cattle in the Czech Republic: 472.20–665.40 [267] vs. 760.23 μkat∙L^−1^ of whole blood [313]). According to Esworthy et al. [347], a quantitative determination of protein or DNA content for the standardization of analytical data used to express GPx enzymatic activity, is necessary.

The interpretation of GPx activity values is also affected by the stage of reproductive cycle of the animal (e.g., estrous cycle phase, pregnancy) and the selected reference unit. Different SeGPx activity profiles per mg of hemoglobin vs. mL of whole blood, or per mL of blood plasma vs. mg of blood plasma protein during peri-oestrus period in sows reflect metabolic changes induced by the ovulation process and with this associated conditions for oxidative stress, which can play a role in the change of enzymatic activities [345]. Likewise, different SeGPx profiles were obtained (mL of blood plasma vs. blood plasma protein—expressed per unit of blood plasma protein) in sows during early gestation. This is probably due to a decrease in blood plasma protein concentration during the first month of gestation, most likely due to a decrease in plasma albumin concentration [348].

It follows from the above that while for the lower limit of the physiological range of selenium in whole blood of cattle a more uniform standard is used (50–100 μg∙L^−1^), the unified reference limit for GPx activity practically does not exist. Lacking standardization in determining GPx activity and interpretation of results leads to the study of this issue. It is recommended that each laboratory establishes its own regression equation [269].

GPx activity in serum or whole blood shows a similar time relation, with regard to supplementation, as selenium level (in serum vs. whole blood), i.e., serum activity reflects short-term supplementation and whole blood activity of previous supplementation levels [349]. Approximately 98% of GPx activity in peripheral blood is associated with erythrocytes and about 73% of selenium in blood is contained in the cellular component of the blood [350]. The life span of erythrocytes in adult cattle is approximately 160 days [351]. Bovine erythrocytes contain only selenium-dependent GPx activity [352], therefore selenium concentrations in blood or erythrocytes and GPx activity in blood or erythrocytes are excellent indicators of long-term selenium status in cattle [307].

Dalto et al. [353] observed that SeGPx activity response in blood plasma on selenium supplementation during the peri-oestrus period was more pronounced under similar experimental conditions than in whole blood [334]. Such influence of blood fractions cannot be seen in long-term profiles of SeGPx activity, SeGPx in blood plasma is therefore a better indicator of acute response to oxidative stress. The main metabolic changes induced by the physiological state of the animal may play an important role in the homeostasis of this enzyme [345].

The SeGPx activity value also depends on the estrous phase of females [345]. In the longer term, it affects the SeGPx activity as well as the level of vitamin B_6_ [353,354,355], which plays a central role in amino acid metabolism. B_6_-dependent enzymes catalyze most reactions of the transsulfuration pathways, ensuring the conversion of homocysteine to cysteine and further into GPx proteins. Because mammals metabolize sulfur- and seleno-amino acids similarly, vitamin B_6_ plays an important role in the fate of sulfur-homocysteine and its selenium counterpart between transsulfuration and one-carbon metabolism, especially in conditions of oxidative stress. This is extremely important in reproduction because ovarian metabolism can generate excessive amounts of ROS during a peri-estrus period that can disturb ovarian function and early embryo development. At a later stage of gestation, placentation increases embryo oxygen tension and may cause higher expression of ROS markers and lead to embryo death. Metabolic accumulation ROS surprisingly positively regulates the flow of one-carbon units to transsulfuration and negatively regulates remethylation. However, in the embryos, the transsulfuration pathway is not functional [355].

The GPx activity in samples is most often measured by the method of Paglia and Valentine [356]—([102,267,271,303,306,342,344,346,357,358,359,360]) (Appendix Table A1), which is based on the measurement of the decrease in light absorbance at 340 nm after oxidation of glutathione by cumene hydroperoxide catalyzed by glutathione peroxidase [306].

Besides the detection of GPx activity in various tissues and organs (whole blood [270], serum [48], liver [357,361], testes [362], skin [102]), various types of this enzyme [(erythrocyte GPx [304], GPx1 [303], GPx3 [360], GPx4 [363] (Appendix Table A1)] are also detected. The clinical relevance of determining the activity of different types of GPx in various tissues is very different. As a selenium status marker, glutathione peroxidase 3 (GPx3) [364], a glycosylated protein secreted to extracellular compartments [365], is also often used as a selenium status marker [364]. It uses a wide range of substrates—H_2_O_2_, fatty acid hydroperoxides and phospholipid hydroperoxidases, and is an effective antioxidant of blood plasma [365].

The activity of erythrocyte GPx in sheep is the dominating component (97.3%) of whole blood GPx activity; on the contrary, the proportion of serum GPx3 activity (2.7%) has a very low significance [366].

Cellular GPx (GPx1) and extracellular GPx (GPx3) are expressed in the liver, heart, placenta, gastrointestinal tract (GIT), thyroid gland, kidneys and erythrocytes in many species, including human, rat and mouse [56,367]. GPx1 and GPx3 are also expressed in bovine mammary epithelial cells [368]. In addition to *GPx1* and *GPx3*, *GPx4* mRNA was also detected in bovine mammary tissue [369]. Enzyme GPx2 is found primarily in the GIT, on the contrary, in the mammary gland, its amount is very low [370].

## 4. Dietary Addition of Selenium

### 4.1. Intake Recommendations for Selenium in Animals

An adequate dietary selenium intake helps to prevent disease caused by its deficiency, prevents the accumulation of lipid hydroperoxides in organs and tissues and thus protects them from damage by ROS [371,372,373]. The daily recommended dose of selenium for the animals is shown in Table 1. When determining the dose, the presence of some other food components that antagonize selenium, e.g., sulfur (S), should be taken into account. Higher dietary sulfur intake reduces plasma selenium concentration and its bioavailability in the organism [374]. Other antagonistic relationships have been described between selenium and other essential or toxic elements: As, Cu, Ni, Co, Cr, Mn, Zn, Cd, Sn, Pb, Hg, Bi, Mo, Ag, Au [375].

### 4.2. Dietary Forms of Selenium

Besides traditional forms of selenium—inorganic (selenite [308,316,385,386,387,388], selenate [387]), organic (selenomethionine [387,389], dimethylselenide, and others [387]), in the form of selenized yeasts [277,316,386,388,390] or selenium bound to *Chlorella* algae biomass [391,392], the use of its nanoform, which substantially increases its biological utilization in the organism, has recently come to the forefront of interest.

## 5. Conclusions

Selenium is an important essential element that interferes through selenoproteins in many physiological processes of the organism and affects the production and reproductive properties of mammalian livestock. By adequate supply of selenium in the feed, it can effectively prevent health problems from its deficiency. Knowledge of the importance of selenium in the body is not yet sufficiently comprehensive, and even less so in animal species, and a deeper study of the effects of selenium may reveal a number of new biologically significant processes.

## Figures and Tables

**Table 1:** Recommended daily intake of selenium for animals.

Animal Species	RDI of Se	Reference
pigs	0.15–0.30 mg∙kg^−1^ of feed	[123]
beef cattle (*)	100 μg∙kg^−1^ of DM of feed	[376,377]
dairy cattle	300 μg∙kg^−1^ of DM of feed	[376,377]
cattle—calves	100 μg∙kg^−1^ of DM of feed	[376,377]
sheep	0.1–0.2 mg∙kg^−1^ of DM of feed	[378]
goats	0.1 mg∙kg^−1^ of DM of feed	[379]
horses	0.1 ppm of DM of feed for idle horses	[380,381]
	0.3 ppm of DM of feed for exercising horses	[380,381]
donkeys	~2 mg∙day^−1^ 0.1–0.15 mg∙100 kg^−1^ BW	[382] [380]

RDI—recommended daily intake; DM—dry matter; (*) hypermuscular breeds (e.g., Belgian Blue) 300 μg∙kg^−1^ of DM of feed [73,383]; BW—body weight. The maximum tolerable level of Se in the feed (mg∙kg^−1^ of DM): pigs—4, cattle, sheep, goats, horses and donkeys—5 [384].

## Abbreviations

AAS	atomic absorption spectroscopy
ABTS^+^	2,2′-azino-bis(3-ethylbenzothiazoline-6-sulphonic acid)
ADF	acid detergent fiber
AdoHcy	S-Adenosyl-homocysteine
*Aebp2*	AE binding protein 2 gene
ALP	alkaline phosphatase
aNDF	amylase-treated neutral detergent fiber
ApoER2	apolipoprotein E receptor 2
AST	aspartate aminotransferase
ATP	adenosine triphosphate
Bax	pro-apoptotic protein Bax, Bcl-2-associated X protein
Bcl-2	anti-apoptotic protein Bcl-2 (B-cell lymphoma 2)
BHT	butylated hydroxytoluene
BW	body weight
Caco-2	caco-2 cell line—heterogeneous human epithelial colorectal adenocarcinoma cells
CAT	catalase
*CAT*	catalase gene
cGPx	cellular glutathione peroxidase; GPx1
CH_3_Se	methylselenol
CNS	central nervous system
Cu	copper
Cu/Zn-SOD	copper/zinc superoxide dismutase; SOD1
D1,2,3	deionidase 1,2,3
DM	dry matter
DNA	deoxyribonucleic acid
DTNB	5-5′-dithiobis[2-nitrobenzoic acid]
ECG	electrocardiography
EDTA	ethylenediaminetetraacetic acid
ELISA	enzyme-linked immunosorbent assay
ESCs	embryonic stem cells
Ex/Em	excitation/emission
FIA-GF-AAS	flow injection analysis-graphite furnace-atomic absorption spectrometry
FOX	xylenol orange
GDH	glutamate dehydrogenase
GGT	gamma-glutamyl transferase
GH	growth hormone
GIT	gastrointestinal tract
GPx	glutathione peroxidase
GPx1,2,3,4	glutathione peroxidase 1,2,3,4
*GPx1,2,3,4*	glutathione peroxidase 1,2,3,4 genes
GR	glutathione reductase
Grx	glutaredoxin
GSH	reduced glutathione
GS-SeH	glutathioselenol
GSSG	oxidized glutathione
GS-TNB	glutathione adduct of GSH
Hb	hemoglobin
HCl	hydrochloric acid
Hcy	homocysteine
HDAC	histone deacetylase
Hg	mercury (*hydrargyrum)*
HG-AAS	hydride generation-atomic absorption spectrometry
HNO_3_	nitric acid
H_2_O_2_	hydrogen peroxide
HPLC	high-performance liquid chromatography
HPLC-ICP-MS	high-performance liquid chromatography-inductively coupled plasma-mass spectrometry
HSe^−^	hydrogen selenide ion
H_2_Se	hydrogen selenide
HT	hematein
HTH_2_	hematoxylin
ICP	inductively coupled plasma
ICP-MS	inductively coupled plasma-mass spectrometry
ICP-OES	inductively coupled plasma-optical emission spectrometry
IEC	ion exchange chromatography
IGF-1	insulin-like growth factor 1
IGF-1R	insulin-like growth factor 1 receptor
INT	2-(4-iodophenyl)-3-(4-nitrophenol)-5-phenyltetrazolium chloride
IRE	iron responsive element
IRP	iron regulatory protein
LC	liquid chromatography
LC-ICP-MS	liquid chromatography-inductively coupled plasma-mass spectrometry
LR	linear regression
MCSeP	mitochondrial capsule selenoprotein
MDA	malondialdehyde
MDA-TBA_2_	adduct formed by reaction of MDA with TBA
MeSeCys	Se-methylselenocysteine
Met	methionine
min–max	minimum‒maximum
miRNA	microRNA—a small non-coding RNA molecule
Mn	manganese
MnSOD	manganese superoxide dismutase; SOD2
mRNA	messenger ribonucleic acid
MTs	metallothioneins
*MT-I*	metallothionein-I gene
*MT-II*	metallothionein-II gene
NaBH_4_	sodium borohydride
NAD	nicotinamide adenine dinucleotide
NADP^+^	nicotinamide adenine dinucleotide phosphate
NADPH	reduced form of NADP^+^
NaOH	sodium hydroxide
Na_2_SeO_3_	sodium selenite
NBT	nitroblue tetrazolium
NH_3_	ammonia
NMD	nutritional muscular dystrophy
O_2_	dioxygen
O_2_^−^	superoxide anion
OD	optical density
OS	oxidative stress
*p53*	*p53* gene encoding the tumor suppressor protein *p53*
PCV	packed cell volume
PDGF-A	platelet-derived growth factor A
PDGF-B	platelet-derived growth factor B
pH	potential of hydrogen (*pondus hydrogenia*)
PHGPx	phospholipid-hydroperoxide GPx
PI3K/Akt pathway	phosphatidylinositol 3′-kinase/protein kinase B (serine/threonine-protein kinase) pathway
PMN	polymorphonuclear leucocytes
PO_4_^3−^	phosphate ion
PR	polynomial regression
*PRDX1–6*	peroxiredoxin 1–6 genes
*Prickle2*	prickle homolog 2 gene
PUFAs	polyunsaturated fatty acids
*r*	correlation coefficient
*R^2^, r^2^*	coefficient of determination (*r^2^* for simple linear regression)
RDI	recommended daily intake
RNA	ribonucleic acid
*Rnd2*	Rho family GTPase (guanosine triphosphatase) 2 gene
ROH	lipid hydroxide
ROOH	lipid hydroperoxide
ROS	reactive oxygen species
RPC	reversed-phase chromatography
RTK	receptor tyrosine kinase
RV	reference value
S	sulfur
SAM	S-adenosylmethionine
SCC	somatic cell count
SCFAs	short-chain fatty acids
SCS	somatic cell score
SD	standard deviation
SE	standard error
Se	selenium
Se^0^	elemental selenium
Se^+IV^	selenite
Se^+VI^	selenate
SECIS	selenocysteine insertion sequence
SeCys	selenocysteine
SeCys2	selenocystine
SeGPx	selenium-dependent glutathione peroxidase
SeH_4_	tetrahydridoselenonium dication
*SELENBP1*	selenium binding protein 1 gene
SEM	standard error of mean
SeMet	selenomethionine
SeO_3_^2−^	selenite
SeO_4_^2−^	selenate
*SEP15*	selenoprotein 15 gene
SEPP	selenoprotein P
*SEPP1*	selenoprotein P gene
SO_4_^2−^	sulfate ion
SOD	superoxide dismutase
SOD1,2,3	superoxide dismutase 1,2,3
*SOD1,2,3*	superoxide dismutase 1–3 genes
SPS-2	selenophosphate synthase-2
*SPS-2*	selenophosphate synthase-2 gene
T3	triiodothyronine
T4	thyroxine
TBA	thiobarbituric acid
TBARS	thiobarbituric acid reactive substances
TBH	tertiary butyl hydroperoxide
TNB	5-thio-2-nitrobenzoic acid
Trx	thioredoxin
*TRXNRD1–2*	thioredoxin reductase 1/2 genes
TrxR	thioredoxin reductase
TSP	transsulfuration pathway
UGA	nucleotide triplet UGA encoding selenocysteine
3′ UTR	3′ untranslated region
UV	ultraviolet
VESD	vitamin E/selenium deficiency
VFA	volatile fatty acid
VG-ICP-MS	vapor generation-inductively coupled plasma-mass spectrometry
WMD	white muscle disease
x	mean
XOD	xanthine oxidase
Zn	zinc

## Appendix A

**Table A1:** Values of selenium concentration, GPx activity and oxidative stress parameters (SOD, CAT, MDA) in animals in different research studies, or their reference values.

Animals	Selenium Concentration	GPx Activity	SOD Activity	CAT Activity	MDA Level	Reference
Specification (Region, Breed, Sex, Age, Weight)	Units	Units	Units	Units	Units	
**Pigs**						
---	RV: 0.12–0.30 μg∙mL^−1^ (in serum)	RV: 100–200 μmol∙min^−1^ at 37 °C∙g^−1^ Hb (erythrocyte GPx)	---	---	---	[304]
Pigs - age of < 1 day - 1–9 days - 10–29 days - 30–70 days - 71–180 days - 181–300 days - 301–700 days - > 700 days	RV: 70–90 70–120 70–120 100–160 140–190 180–220 180–220 180–220 ng∙mL^−1^ (in serum)	---	---	---	---	[314]
7-day-old piglets (Duroc × Landrace)—control group	---	~222 U∙mg^−1^ protein (in liver) *1	~265 U∙mg^−1^ protein (in liver) *1	---	~2.4 nmol∙mg^−1^ protein (in liver) *1	[361]
Piglets from crossbred pregnant sows (Large White × Landrace) on day 107 of gestation—control animals	---	621.69 ± 24.93 mmol∙L^−1^ (x ± SEM) (in serum) *2	---	7.38 ± 0.27 U∙mL^−1^ (x ± SEM) (in serum) *2	---	[343]
Crossbred (Yorkshire × Landrace × Duroc) weaned pigs (28 ± 2 days of age)	---	0.13 U∙g^−1^ Hb (erythrocyte GPx) *3	443.3 U∙g^−1^ Hb (erythrocyte Cu/Zn-SOD) *3	1.74 U∙g^−1^ Hb (erythrocyte CAT) *3	4.29 μM (in plasma) *3	[342]
**Cattle**						
---	RV: 0.08–0.30 μg∙mL^−1^ (in serum)	RV: 19–36 μmol∙min^−1^ at 37 °C∙g^−1^ Hb (erythrocyte GPx)	---	---	---	[304]
Cattle - age of <1 day - 1–9 days - 10–29 days - 30–300 days - 301–700 days - >700 days	RV: 50–70 50–70 55–75 60–80 65–90 70–100 ng∙mL^−1^ (in serum)	---	---	---	---	[314]
Holstein-Frisian cows 12 h postpartum—control group	129.0 ± 18.0 ng∙mL^−1^ (x ± SD) (in blood) *4	90.6 ± 16.1 μkat∙L^−1^ (x ± SD) (in whole blood) *4	---	---	5.71 ± 0.94 μM (x ± SD) (in serum) *4	[270]
Cattle—control group	---	172.5 ± 30.7 U∙g^−1^ Hb (x ± SD) (erythrocyte GPx); 24.3 ± 4.8 U∙g^−1^ protein (x ± SD) (hepatic GPx) *5	---	---	---	[357]
Cattle (dairy cows, bulls, heifers) in Czech Republic	78.25 ± 46.67 (1.33–212.40) μg∙L^−1^ (x ± SD; min–max) (in whole blood) *6	525.51 ± 335.56 (0.41–1521.1) μkat∙L^−1^ (x ± SD; min–max) (in whole blood) *6 RV of GPx activity: 472.20–665.40 * μkat∙L^−1^	---	---	---	[267]
Cattle—(a) bulls (b) heifers (c) cows	56.9 ± 43.2 39.0 ± 20.8 83.2 ± 20.0 μg∙L^−1^ (x ± SD) (in whole blood) *6	368.7 ± 343.4 227.4 ± 130.8 741.7 ± 233.5 μkat∙L^−1^ (x ± SD) (in whole blood) *6 RV of GPx activity: 760.23 ** μkat∙L^−1^	---	---	---	[313]
**Sheep**						
---	RV: 0.08–0.50 μg∙mL^−1^ (in serum)	60–180 μmol∙min^−1^ at 37 °C∙g^−1^ Hb (erythrocyte GPx)	---	---	---	[304]
Sheep - age of < 1 day - 1–9 days - 10–29 days - 30–70 days - 71–180 days - 181–300days - 301–700 days - >700 days	RV - 50–80 - 60–90 - 70–100 - 80–110 - 80–110 - 80–110 - 90–120 - 120–160 ng∙mL^−1^ (in serum)	---	---	---	---	[314]
Iranian fat-tailed sheep	---	RV: 191.67–196.52 U∙g^−1^ Hb (in blood) *** *7	RV: 948.65–1011.50 U∙g^−1^ Hb (in blood) *** *7	RV: 1834.29–1915.63 U∙g^−1^ Hb (in blood) *** *7	RV: 0.53–0.60 μmol∙L^−1^ (in blood) *** *7	[346]
Sheep in the Czech Republic (Suffolk or Merinolandschaft breeds)	123.42 ± 57.84 μg∙L^−1^ (x ± SD) (in blood) *8	814.34 ± 463.64 μkat∙L^−1^ (x ± SD) (in blood) RV: >637 μkat∙L^−1^ (LR), resp. > 677 μkat∙L^−1^ in whole blood (PR) **** *8	---	---	---	[271]
Grazing ewes in Serbia (Wirtenberg × Cigaja crossbred sheep)—control group	---	157.4 ± 61.9 μkat∙L^−1^ (in whole blood) *9	---	---	---	[393]
½ Dorper (♂) × ½ Small thin-tailed (♀) crossed ram lambs (4 months old, 25 ± 1 kg) (a) in free-range conditions (b) in individual stalls	---	(a) 84.01 ± 4.33 (b) 71.56 ± 2.06 U∙mg^−^^1^ (x ± SEM) (GPx4 in testes) *10	(a) 6.05 ± 0.03 (b) 5.88 ± 0.12 U∙mg^−^^1^ (x ± SEM) (in testes) *10	(a) 5.28 ± 0.11 (b) 4.29 ± 0.08 U∙mg^−^^1^ (x ± SEM) (in testes) *10	(a) ~0.65 (b) ~1.2 nM∙mg^−^^1^ (in testes) *10	[363]
Akkaraman sheep, weight 20–25 kg, age 6–12 months—control group	---	18.71 ± 1.11 U∙mg ^−^^1^ protein (x ± SD) (in liver) *11	5.00 ± 0.21 U∙mg^−^^1^ protein (x ± SD) (Cu/Zn-SOD in liver) *11	849.24 ± 23.83 k∙g^−^^1^ (x ± SD) (in liver) *11	45.26 ± 1.15 nmol∙g ^−^^1^ (x ± SD) (in liver) *11	[394]
**Goats**						
Goats - age of <1 day - 1–9 days - 10–29 days - 30–70 days - 71–180 days - 181–300 days - 301–700 days - >700 days	RV - 50–80 - 60–90, - 70–100 - 80–110 - 80–110 - 80–110 - 90–120 - 120–160 ng∙mL^−1^ (in serum)	---	---	---	---	[314]
Red Sokoto goats of about 1-year-old, weighing 10–14 kg—control group	---	~54 IU∙L^−1^ (in serum) *12	~2.4 IU∙L^−1^ (in serum) *12	~47.4 IU∙L^−1^ (in serum) *12	~ 1.25 nmol∙L^−1^ (in serum) *12	[344]
Weanling Boer goat bucks (2 months old) from selenium deficiency region in central China—control group	0.6491 mg∙kg^−1^ (in testes) *13	13.55 ± 3.15 U∙mL^−1^ (x ± SD) (in semen); 65.20 ± 5.89 U∙mg^−1^ (x ± SD) (testicular GPx) *13	---	---	---	[362]
Cashmere goats, aged 3-year-old and weighing 34.35 ± 0.94 kg from selenium deficiency region in China—control group	85.24 ng∙mL^−1^ (in serum); 32.6 ng∙mL^−1^ (in skin) *14	264.82 U∙ml^−1^ (in serum); 113.89 U∙mL^−1^ (in skin) *14	72 U∙mL^−1^ (in serum); 9.29 U∙mL^−1^ (in skin) *14		2.31 nmol∙ml^−1^ (in serum) 0.46 nmol∙ml^−1^ (in skin) *14	[102]
**Horses**						
---	RV: 0.14–0.25 μg∙mL^−1^ (in serum)	RV: 30–150 μmol∙min^−1^ at 37 °C∙g^−1^ Hb (erythrocyte GPx)	---	---	---	[304]
Horses - age of < 1 day - 1–9 days - 10–29 days - 30–70 days - 71–180 days - 181–300 days - 301–700 days - >700 days	RV: 70–90 70–90 80–110 90–110 90–110 90–110 100–130 130–160 ng∙mL^−1^ (in serum)	---	---	---	---	[314]
Arabian mares—healthy (control group), age of 15 ± 1.5 months	---	32.07 ± 5.10 U∙g^−1^ Hb (x ± SE) (erythrocyte GPx) *15	---	---	1.50 ± 0.13 nmol∙mL^−1^ (x ± SE) (in plasma) *15	[395]
Standardbred horses (mares, geldings)—control group	~0.052 ppm (in plasma); 0.15 ppm (in red blood cells) *16	~ 100 U∙g^−1^ Hb (in whole blood) *16	---	---	---	[306]
Polish Sztumski, Polish Lidzbark, and Sokolski horses (geldings and mares), age: 4–10 years	---	36 ± 14 (9–67) U∙g^−1^ Hb (x ± SD; min–max) *17	---	---	---	[359]
Italian Saddle horses from herd in Piacenza province (Italy), age: 13.6 ± 4.8 years—control group	174.7 ng∙g^−1^ (in blood); 87.7 ng∙g^−1^ (in plasma) *18	23,085 U∙L^−1^ 178.0 U∙g^−1^ Hb (GPx1 in blood); 839.6 U∙L^−1^ (GPx3 in plasma) *18	---	---	---	[303]
Horses under maintenance care (females, Arabians, ~380 kg, ~14 years) and athlete animals (both genders, Mangalarga Marchador, ~365 kg, ~7 years)—values before test	---	328.37 ± 10.29 UL∙g^−1^ Hb (x ± SD) (in blood) *19	1983.05 ± 140.84 UL∙g^−1^ Hb (x ± SD) (in blood) *19	---	---	[396]
Slovenian warm-blooded horses (both genders), age of 2–10 years, body weight of 389.7 ± 126.1 kg	---	53.2 ± 1.4 U∙g^−1^ Hb (x ± SE) (in whole blood) *20	1330.3 ± 20.8 U∙g^−1^ Hb (x ± SE) (in whole blood) *20	---	---	[358]
Arabian mares (4–6 years old)—control group	---	---	---	---	1.006 ± 0.078 (0.870–1.100) μmol∙L^−1^ (x ± SD; min−max) (in blood) *21	[397]
Male Arabian horses (4–6 years old)—control (healthy) group	---	---	110.00 ± 6.26 U∙mL^−1^ (x ± SE) (in erythrocyte hemolysate) *22	1480.66 ± 543.00 U∙mL^−1^ (x ± SE) (in erythrocyte hemolysate) *22	1.00 ± 0.12 μmol∙L^−1^ (x ± SE) (in erythrocyte hemolysate) *22	[398]
Standardbreds trotters (mares, stallions), age 16–20 months—healthy animals	---	51.2 ± 1.93 U∙g^−1^ Hb (x ± SEM) (in whole blood) *23	---	---	---	[399]
18-month-old horses (fillies, geldings) of American Quarter Horse, American Paint Horse, and grade-stock type horses—control group	0.108 μg∙mL^−1^ (in plasma)	10.0 mU∙mg^−1^ protein (GPx3 in plasma); 233 mU∙mg^−1^ Hb (GPx1 in red blood cells) *24	---	---	---	[360]
**Donkeys**						
Female donkeys, 2–5 years of age and 130–190 kg in weight—control group	120.62 ± 4.07 (mg∙kg^−1^) (x ± SEM) (in serum) *25	---	---	---	---	[305,400]

SOD—superoxide dismutase; RV—reference value; Hb—hemoglobin; x ± SE/SD/SEM—mean ± standard error/standard deviation/standard error of mean; min‒max—minimum‒maximum; ~—the value was subtracted from the graph. * The references values of GPx activity (*y*) were calculated for the use in diagnosis of insufficient selenium in cattle in the Czech Republic as equivalent to reference range of selenium concentration in whole blood of cattle 70–100 μg∙L^−1^ according to the regression equation (*x*—whole blood selenium concentration): *y =* 6.44*x +* 21.40. ** The references value of GPx activity (*y*) was calculated as equivalent to reference value of selenium concentration in whole blood of cattle 100 μg∙L^−1^ according to the regression equation (*x*—whole blood selenium concentration): *y =* 8.29*x* − 68.77. *** All reference values were determined for Iranian fat-tailed sheep. **** The references values of GPx activity (*y*) for sheep breeded in the Czech Republic were calculated as equivalent to selenium concentration in whole blood of sheep 100 μg∙L^−1^ according to the following regression equations (*x*—whole blood selenium concentration): *y =* 7.5857*x* − 121.87 (linear regression; LR) or *y =* −0.0167*x*^2^ *+* 11.993*x* − 355.57 (polynomial regression; PR). *1—colorimetric methods according to the commercial kits (Nanjing Jiancheng Bioengineering Institute, Nanjing, Jiangsu, China)—**GPx:** by measuring the reduction of glutathione per min after the subtraction of the nonenzymatic reaction; **SOD:** using the hydroxylamine method, absorbance was recorded at 550 nm; **MDA:** enzymatic colorimetric method according to the commercial kit (Nanjing KeyGEN BioTech, Nanjing, China) using the thiobarbituric acid (TBA) method to generate a colored product with an absorbance at 532 nm. *2—**GPx, CAT:** colorimetric methods using assay kits from the Nanjing Jiancheng Bioengineering Institute, Nanjing, Jiangsu, China. **GPx**—see *1; **CAT**—the original methology was not verified. *3—commercially available kits (Randox, Antrim, England)—**GPx:** the method is based on that of Paglia and Valentine [356]: GPx catalyzes the oxidation of glutathione (GSH) by cumene hydroperoxide, in the presence of glutathione reductase (GR) and NADPH the oxidized glutathione (GSSG) is immediately converted to the reduced form with a concomitant oxidation of NADPH to NADP^+^, the decrease in absorbance at 340 nm is measured; for a detailed description of the method, see *5. SOD: the method is based on a red formazan dye formation: it employs xanthine and xanthine oxidase (XOD) to generate superoxide radicals which react with 2-(4-iodophenyl)-3-(4-nitrophenol)-5-phenyltetrazolium chloride (INT) to form a red formazan dye. **CAT:** the method described by Aebi [401]—the original methology was not verified; **MDA:** the method according to the procedure of the ABTS^+^ (2,2′-azino-bis(3-ethylbenzothiazoline-6-sulphonic acid) scavenging assay according Yagi [402] with modifications described by Augustin et al. [403]: the susceptibility of plasma to copper-induced lipid oxidation was determined by measuring thiobarbituric acid-reactive substances (TBARS) as MDA concentrations. The fluorescence of the supernatant was assayed with a fluorometer (excitation = 532 nm, emission = 553 nm). *4—**Se:** HG-AAS, measuring of absorption at 196 nm was performed after SeH_4_ formation in the hydride system with NaBH_4_ and NaOH. **GPx:** the samples were hemolyzed using Drabkin’s reagent. **GPx** present in the samples reduces tertiary butyl hydroperoxide (TBH), glutathione (GSH) as the donor of hydrogen becomes oxidized to GSSG, in the second phase of this coupled reaction GSSG is reduced to GSH by NADPH and glutathione reductase (GR), the low concentration of TBH (under 2.32 mM) as used in this method, determines only the activity of SeGPx, the reduction of NADPH was measured at 366 nm. **MDA:** using orthophosphoric acid, thiobarbituric acid and hydrated ferrous sulfate solution, the produced chromogen was extracted with n-butyl alcohol, the butanol layer was separated for spectrophotometric measurement at 535 nm. *5—**Erythrocytic GPx** and **hepatic GPx:** using test kits supplied by Oxis Research (Bioxytech^®^ GSH-PX-340) U.S.A. The GPx-340™ assay is an indirect measure of the activity of cGPx (cellular glutathione peroxidase) [356]. Principle of the procedure: Oxidized glutathione (GSSG), produced upon reduction of an organic peroxide by cGPx, is recycled to its reduced state by the enzyme glutathione reductase (GR): cGPx: R − O − O − H + 2GSH → R − O − H + GSSG + H_2_O; GR: GSSG + NADPH + H^+^ → 2GSH + NADP^+^. The oxidation of NADPH to NADP^+^ is accompanied by a decrease in absorbance at 340 nm (A_340_) providing a spectrophotometric means for monitoring GPx enzyme activity. The molar extinction coefficient for NADPH is 6220 M^−1^∙cm^−1^ at 340 nm. To assay cGPx, a cell or tissue homogenate is added to a solution containing glutathione, glutathione reductase, and NADPH. The enzyme reaction is initiated by adding the substrate, tert-butyl hydroperoxide, and the A_340_ is recorded. The rate of decrease in the A_340_ is directly proportional to the GPx activity in the sample. *6**—Se:** HG-AAS; **GPx:** the method developed by Paglia and Valentine [356], using the set supplied by Randox. *7—**GPx:** the method of Paglia and Valentine [356], using RANSEL Kit, (Randox, UK), see *3; **SOD:** a modified method of iodophenyl nitrophenol phenyltetrazolium chloride (RANSOD Kit, Randox, UK), see *3; **CAT:** the method of Beers and Sizer [404], using the ferrous oxidation in xylenol orange (FOX) assay. Samples containing CAT were incubated with H_2_O_2_ for varying time intervals prior to rapid mixing of aliquots of the incubation mixtures with FOX reagent, which measures residual H_2_O_2_, absorbance was read at 560 nm. **MDA:** the thiobarbituric acid method was used to quantitate MDA-reactive products Plaser and Cushman [405], TBA and MDA react to form a schiff base adduct under high temperature/acidic conditions to produce a chromogenic/fluorescent product that can be easily measured employing various analytical techniques such as spectrophotometric or fluorometric methods. *8—**Se:** using HG-AAS according to the method described by Pechova et al. [272]. **GPx:** the method developed by Paglia and Valentine [356], using a set supplied by Randox. *9**—GPx:** the blood samples were hemolysed in Drabkin’s reagent, GPx activity was analyzed spectophotometrically by the coupled test [406] using tertiary butyl hydroperoxide (TBH) below 2.32 mM in order to measure only the activity of selenium dependent GPx [407]. *10**—GPx, SOD, CAT** and **MDA:** ELISA kits from Beijing SINO-UK Institute of Biological Technology (Beijing, China): glutathione peroxidase 4 (GPx4, No. HY-60005), superoxide dismutase (SOD, No. HY-60001), catalase (No. HY-60015), and MDA (HY-60003). The methods were not verified. *11**—GPx, Cu/Zn-SOD:** by the use of commercially available kits (Randox Laboratory, Crumlin, Ireland); **CAT:** was determined according to Aebi’s method; **MDA:** was determined by the method according to Jain et al. [408]. *12—The GPx, SOD, CAT and MDA assay protocols were based on methods described by Paglia and Valentine [356], Martin et al. [409], Aebi [401] and Janero [410], respectively. **GPx, SOD, MDA:** using assay kits: NWK-GPx01, NWK-SOD02, and NWK-MDA01, respectively, purchased from Northwest Life Science Specialties, Vancouver, Canada. **CAT:** the activity was evaluated using catalase kit, purchased from Abcam PLC, 330 Cambridge Science Park, UK. **GPx:** The NWLSS™ Glutathione Assay is a modification of the method first described by Tietze [411]. The general thiol reagent, 5-5′-dithiobis [2-nitrobenzoic acid] (DTNB, Ellman’s Reagent) reacts with GSH to form the 412 nm chromophore, 5-thio-2-nitrobenzoic acid (TNB) and GS-TNB (glutathione adduct of GSH). The GS-TNB is subsequently reduced by glutathione reductase and β-nicotinamide adenine dinucleotide phosphate (NADPH), releasing a second TNB molecule and recycling GSH; thus amplifying the response. Any oxidized GSH (GSSG) initially present in the reaction mixture or formed from the mixed disulfide reaction of GSH with GS-TNB is rapidly reduced to GSH. **SOD:** The NWLSS™ NWK-SOD02 method is based on monitoring the auto-oxidation rate of hematoxylin (HTH_2_) (which is converted to hematein - HT) as originally described by Martin et al. [409], with modifications to increase robustness and reliability. Briefly, in the presence of SOD enzyme at specific assay pH, the rate of auto-oxidation is inhibited and the percentage of inhibition is linearly proportional to the amount of SOD present within a specific range. Sample SOD activity is determined by measuring ratios of auto-oxidation rates in the presence and absence of the sample and expressed as traditional McCord Fridovich “cytochrome c” units. The basic principal of the assay is shown schematically by the following equation: O_2_ + HTH_2_ → H_2_O_2_ + HT. **MDA:** The NWK-MDA01 assay is based on the reaction of MDA with TBA forming an MDA-TBA_2_ adduct that absorbs strongly at 532 nm. Butylated hydroxytoluene (BHT) and EDTA are added to the sample and reaction mixture to minimize oxidation of lipids that contribute artifactually during sample processing and the TBA reaction [412,413]. The temperature of the reaction mixture has also been reduced to minimize the decomposition of lipid hydroperoxides. Because much of the MDA protein is bound, mostly as a Schiff base, the pH of the reaction has been optimized to facilitate hydrolysis of the MDA [414]. Additionally, the reaction mixture is subjected to derivative spectrophotometric analysis that resolves the problem of the variable and nonlinear baseline observed when attempting to measure the A_532_ absorbance in various biological samples. **CAT:** Catalase Activity Assay Kit (Colorimetric/Fluorometric) (ab83464, Abcam, Cambridge, MA, USA) is a highly sensitive, simple and direct assay for measuring catalase activity in a variety of biological samples such as cell and tissue lysates or biological fluids. In this assay, the catalase present in the sample reacts with hydrogen peroxide (H_2_O_2_) to produce water and oxygen. The unconverted H_2_O_2_ reacts with the probe to produce a product that can be measured colorimetrically at OD 570 nm or fluorometrically at Ex/Em = 535/587 nm. Therefore, the catalase activity present in the sample is reversely proportional to the obtained signal. The kit can detect as little as 1 μU of catalase activity. *13**—Se:** with the procedure following the fluorometric method by Reaner and Veillon [415] with some modifications, the concentration was determined by atomic fluoro-spectrophotometry. **GPx—**using hydrogen peroxide as a substrate [416]. *14**—Se:** using ASF-230E hydride generation atomic fluorescence spectrometer; **GPx:** according to the procedure of Paglia and Valentine [356] using hydrogen peroxide as a substrate. SOD: using xanthine-xanthine oxidase and nitroblue tetrazolium (NBT) [417], one unit of SOD is defined as the amount of protein that inhibits the rate of NBT reduction by 50%. **MDA:** using the procedure described by Wills [418] as nanomoles of MDA per milligram of protein. *15**—GPx:** spectrophotometry, using cumene hydroperoxide as substrate [419]. Oxide glutathione (GSSG), produced by the action of erythrocyte GPx and cumene hydroperoxide, was reduced by glutathione reductase (GR) and NADPH. The decrease in the concentration of NADPH was measured at 340 nm [419]. **MDA:** spectrophotometry (colorimetric changes at 532 nm with a spectrophotometer), MDA level was measured on the basis of the reaction between MDA and TBA and detecting the colorimetric changes at 532 nm with a spectrophotometer [420,421]. *16**—Se:** AAS; **GPx:** the activity was measured using a commercial assay (Ransel; RANDOX laboratories, Mississauga, Ontario) based on the method by Paglia and Valentine [356], which measures the decrease in absorbance of light at 340 nm when glutathione is oxidized by cumene hydroperoxide catalyzed by glutathione peroxidase. *17**—GPx:** reduction of oxidized glutathione catalyzed by GR with NADP formation and decrease of absorbance at 340 nm using reagents from Bioxytech (cat. nr 21017), OXIS International, Inc., and assay according Paglia and Valentine [356]. *18**—Se in whole blood and in plasma:** HPLC-ICP-MS; **GPx1 in whole blood:** according to the method of Paglia and Valentine [356] using a commercial kit (Ransel kit, Randox), and **GPx3 in plasma:** the previous method. *19**—GPx, SOD:** using commercial kits (Randox^®^—Ransel: Crumlin, County Antrim, UK) and a semi-automatic biochemical analyser. *20**—GPx:** spectrophotometrically with an automated biochemical analyser using the commercial Ransel kit (Randox Laboratories, Crumlin, UK) based on the method of Paglia and Valentine [356]; **SOD:** spectrophotometrically with an automatic biochemical analyser using commercially available Ransod kit (Randox Laboratories, Crumlin, UK) based on the original method of McCord and Fridovich [422]. *21**—MDA:** using commercial ELISA Kits (Cayman Chemical, Ann Arbor, MI, USA). The MDA in the sample reacts with TBA to generate the MDA-TBA adduct. The MDA-TBA adduct can be easily quantified colorimetrically (λ = 532 nm) or fluorometrically (Ex/Em = 532/553 nm). This assay detects MDA levels as low as 1 nmol∙well^−1^ colorimetrically and 0.1 nmol∙well^−1^ fluorometrically. *22**—SOD:** the activity was assayed in erythrocyte hemolysate as described by Nishikimi et al. [423] using commercial available kit (Bio-diagnostic, Kit number SD2520); **CAT:** the activity was assayed in the erythrocyte by the method of Aebi [401] using commercial available kit (Bio-diagnostic, Kit number CA2516); **MDA:** the level was determined on the base of MDA reacted with TBA at 532 nm, according to Ohkawa et al. [424] using commercially supplied kits (Bio-diagnostic, Kit number MD2529). *23**—GPx:** Kit Bioxytech cGPX-340 by OXIS Research, Portland, OR, USA. *24**—Se:** using a semiautomated fluorometric technique [425] with the modifications of Beilstein and Whanger [426]; **GPx:** the activities of plasma GPx3 and red blood cell GPx1 were determined by the method of Paglia and Valentine [356], using a Bioxytech GPx-340 Assay Kit (OXIS Research, Portland, OR, USA). The assay provides an indirect measure of GPx activity. The sample to be assayed for GPx was added to a solution containing glutathione (GSH), glutathione reductase, and NAD phosphate (NADPH), to which tertbutyl hydroperoxide was added. Sample GPx catalyzed the reduction of tert-butyl hydroperoxide, using reducing equivalents from GSH yielding oxidized glutathione. Oxidized glutathione was recycled back to GSH by glutathione reductase, using reducing equivalents from NADPH. The consumption of NADPH was accompanied by a decrease in absorbance at 340 nm. The change in NADPH concentration upon initiation of the reaction just described was directly proportional to GPx activity (i.e., 1 mU GPx activity∙mL^−1^ is equivalent to a decrease of 1 nmol of NADPH mL∙min^−1^). The NADPH concentration was calculated using the extinction coefficient (6220 m∙cm^−1^) at 340 nm. *25**—Se:** by using atomic absorption spectroscopy and the commercial kit (Pars Azmoon and Darman Kav, Co., Tehran, Iran).
